# Efflux Pump Overexpression Contributes to Tigecycline Heteroresistance in *Salmonella enterica* serovar Typhimurium

**DOI:** 10.3389/fcimb.2017.00037

**Published:** 2017-02-17

**Authors:** Yi Chen, Daxing Hu, Qijing Zhang, Xiao-Ping Liao, Ya-Hong Liu, Jian Sun

**Affiliations:** ^1^National Risk Assessment Laboratory for Antimicrobial Resistance of Animal Original Bacteria, College of Veterinary Medicine, South China Agricultural UniversityGuangzhou, China; ^2^Guangdong Provincial Key Laboratory of Veterinary Pharmaceutics Development and Safety Evaluation, South China Agricultural UniversityGuangzhou, China; ^3^Department of Veterinary Microbiology and Preventive Medicine, Iowa State UniversityAmes, IA, USA

**Keywords:** tigecycline, *Salmonella enterica* serovar Typhimurium, heteroresistance, efflux pump, plasmid

## Abstract

Bacterial heteroresistance has been identified in several combinations of bacteria and antibiotics, and it complicated the therapeutic strategies. Tigecycline is being used as one of the optimal options for the treatment of infections caused by multidrug-resistant *Salmonella*. This study investigated whether heterorresistance to tigecycline exists in a *Salmonella enterica* serovar Typhimurium strain harboring the *oqxAB*-bearing IncHI2 plasmid pHXY0908. MIC and population analyses were performed to evaluate population-wide susceptibility to tigecycline. The effects of efflux pumps on MIC levels were assessed using the efflux pump inhibitor Phe-Arg-β-naphthylamide, measuring intracellular tigecycline accumulation as well as mRNA levels of regulatory and efflux pump genes. DNA sequencing of regulatory regions were performed and plasmid curing from a resistant strain provided an appropriate control. Results showed that MICs of a parental strain with and without pHXY0908 as well as a plasmid-cured strain 14028/Δp52 were 0.5, 1, and 1 μg/mL, respectively. Population analysis profiling (PAP) illustrated that only the pHXY0908-containg strain was heteroresistant to tigecycline. A fraction of colonies exhibited stable profiles with 4- to 8-fold increases in MIC. The frequencies of emergence of these isolates were higher in the plasmid-containing strain pHXY0908 than either the parental or the 14028/Δp52 strain. Phe-Arg-β-naphthylamide addition restored tigecycline susceptibility of these isolates and intracellular tigecycline accumulation was reduced. Heteroresistant isolates of the strain containing pHXY0908 also had elevated expression of *acrB, ramA*, and *oqxB*. DNA sequencing identified numerous mutations in RamR that have been shown to lead to *ramA* overexpression. In conclusions, heteroresistance to tigecycline in *Salmonella enterica* serovar Typhimurium was manifested in a plasmid-bearing strain. Our results suggest that this phenotype was associated with overexpression of the AcrAB-TolC and OqxAB efflux pumps.

## Introduction

*Salmonella enterica* serovar Typhimurium is an important zoonotic pathogen that leads to various infections in humans and the proliferation of multidrug-resistant isolates, especially those extended-spectrum β-lactamase-producing and fluoroquinolone-resistant strains are of major concerns (Fàbrega et al., 2008). Carbapenems and colistin were therapeutic options for treatment of severe infections caused by multidrug-resistant bacteria such as the Typhimurium serovar (Taneja and Kaur, 2016). However, the emergence of carbapenemase-producing and *mcr-1*-positive *Salmonella* has threatened the treatment options (Liu et al., 2016; Yao et al., 2016).

Tigecycline represents a new class of antibiotic agents, the glycylcyclines. This compound exhibits an extraordinarily broad-spectrum of activity against pathogens, including difficult-to-treat pathogens such as carbapenemase- or extended-spectrum β-lactamases-producing Gram-negative bacteria (Stein and Babinchak, 2013). Antimicrobial activity of tigecycline results from a stacking interaction with nucleobase C1054 within the decoding site of the ribosome and during decoding, tigecycline inhibits the initial codon recognition step of tRNA accommodation (Jenner et al., 2013). Tigecycline is readily transported into the cell and achieves high intracellular concentrations (Ong et al., 2005). This is a key factor when considering treatment of invasive infections caused by multidrug-resistant *Salmonella* (Tang et al., 2016).

Although tigecycline bypasses the classical resistance mechanisms that confer resistance to tetracyclines (Fluit et al., 2005), the compound is still vulnerable to chromosomally encoded efflux pumps (Keeney et al., 2007, 2008; Ruzin et al., 2007). Up-regulation of multidrug efflux pumps such as AcrAB in *Escherichia coli* (Keeney et al., 2008), *Klebsiella pneumonia* (Zhong et al., 2014), and *Enterobacter cloacae* (Keeney et al., 2007); AdeABC in *Acinetobacter baumanni* (Ruzin et al., 2007), and MexXY in *Pseudomonas aeruginosa* (Dean et al., 2003) have all been implicated in tigecycline resistance. An RND family efflux pump, OqxAB may also supplement tigecycline resistance in *K. pneumonia* (Zhong et al., 2014) and *E. cloacae* (Veleba et al., 2013). The AcrAB-TolC efflux pump transports numerous structurally unrelated compounds (Du et al., 2014). Its expression is modulated precisely by its local repressor, AcrR, as well as by global transcriptional regulators of the AraC family, RamA, MarA, SoxS, and Rob (Nikaido et al., 2008; Kehrenberg et al., 2009; Pérez et al., 2012). Mutations in *acrR* cause the loss of its repressor function, resulting in *acrAB* overexpression. In addition, mutations in *ramR, marR*, and *soxR* activate *ramA, marA*, and *soxS*, leading to the upregulation of the *acrAB* efflux pump.

Heteroresistance is the presence of a subpopulation of less susceptible bacteria in a population of fully susceptible bacteria (El-Halfawy and Valvano, 2015). This phenomenon was first described in *Staphylococcus aureus* in 1970 (El-Halfawy and Valvano, 2015) and has been widely investigated. Distinct from the persistence which was linked to phenotypic switching between growing cells and persister cells having reduced growth rates and cells regrown from them remain sensitive to the antibiotic (Balaban et al., 2004), heteroresistance means the coexistence of resistant and susceptible bacterial cells in the same culture (El-Halfawy and Valvano, 2015). Population analysis profiling (PAP) is the conventional method for determining heteroresistance (El-Halfawy and Valvano, 2015). Bacterial antibiotic resistance can be either intrinsic or acquired and these are equally applicable to heteroresistance (El-Halfawy and Valvano, 2015). Heteroresistance can also be an intermediate stage for completely changing from susceptibility to resistance upon exposure of bacteria to an antibiotic (Morand and Mühlemann, 2007; Falagas et al., 2008).

Clinical isolates of *Salmonella* resistant to tigecycline have now been reported (Hentschke et al., 2010). Non-typhoidal *Salmonella* has a wide host specificity and global burden compared to typhoidal *Salmonella* (Smith et al., 2016). Up to this point, there have been no investigations on tigecycline heteroresistance in *Salmonella enterica* serovar Typhimurium. Thus the main purpose of this study was to investigate whether heteroresistance to tigecycline exists in *Salmonella enterica* serovar Typhimurium 14028 and 14028/pHXY0908 (14028 harboring *oqxAB*-bearing IncHI2 plasmid pHXY0908) and to determine molecular mechanisms responsible for tigecycline heteroresistance. 14028/pHXY0908 was obtained by electroporation of a transferable IncHI2-type plasmid pHXY0908 (KM877269) carrying the quinolone resistance determinate *oqxAB* from *S*. Typhimurium-147 into *Salmonella enterica* serovar Typhimurium 14028. The multidrug-resistant IncHI2 plasmid pHXY0908 (GenBank accession number KM877269) from *S*. Typhimurium has been involved in the spread of *oqxAB* in food-producing animals in China (Li et al., 2013).

## Materials and methods

### Strains and antibiotic susceptibility testing

The bacterial strains and plasmids used in this study are listed in Table 1. Typhimurium strains were routinely propagated in Mueller-Hinton (MH) or Luria-Bertani (LB) medium. The MIC of tigecycline was determined by standard broth microdilution methods according recommendations of the Clinical and Laboratory Standards Institute (M100-S25). All media were freshly prepared (<12 h) in order to minimize the oxidative degradation of tigecycline (Hope et al., 2005). The breakpoint criteria used to determine tigecycline phenotype was based on the United States Food and Drug Administration breakpoint criteria [≤ 2 mg/L (susceptibility), 4 mg/L (intermediate), and ≥ 8 mg/L (resistance)]. *E. coli* ATCC 25922 was included and used as a reference strain.

**Table 1:** **Strains and plasmid used in this study**.

**Strain or plasmid**	**Description**
***S*****. TYPHIMURIUM**	
14028	ATCC 14028
14028#2	Derived from 14028 with *ramR* mutation
14028#3	Derived from 14028 with *ramR* mutation
14028/pHXY0908	Derivative of 14028 harboring plasmid pHXY0908
14028/p#36	Heteroresistant isolate of 14028/pHXY0908 with *ramR* mutation
14028/p#52	Heteroresistant isolate of 14028/pHXY0908 with *ramR* mutation
14028/Δp52	Plasmid-cured strain of 14028/p#52
14028/Δp52#17	Resistant isolate of 14028/Δp52
14028/Δp52#18	Resistant isolate of 14028/Δp52
Plasmid	
pHXY0908	An IncHI2-type plasmid containing *oqxAB, sul, aphA1, aadA1, cmlA, aadA2, floR, aac(3′)-IV, aac(6′)-Ib-cr, bla*_*OXA*−1_, *catB3, qacEdelta1* (GenBank accession number KM877269)

### Population analysis profiling (PAP)

PAP was conducted as previously described (Hung et al., 2012). Briefly, 40 μL of overnight culture was subcultured in 4 mL of LB broth, and grown to late logarithmic phase (OD_600_ = 0.3–0.4). Dilutions of 10^−2^–10^−6^ in 0.85% saline were prepared, and 100 μL (~10^8^ bacterial CFU) was spread onto MH agar plates containing tigecycline in serial dilutions at concentrations ranging from 0.125 to 16 μg/mL (0.5 to 16 × MIC). A dilution of 10^−6^ was deposited onto MH plates lacking antibiotic for determination of colony number after 48 h at 37°C. The frequency of appearance of heteroresistant subpopulations was calculated by dividing the number of colonies that grew on antibiotic-containing plates (4–16 × MIC) by the number that grew on antibiotic-free plates (Morand and Mühlemann, 2007). The analysis was performed three times and the mean values of viable CFU were calculated and plotted on a semilogarithmic graph.

### Luria-delbrück fluctuation analysis and stability of resistance profiles

To investigate whether the resistant subpopulations were derived from pre-adapted antibiotic-resistant cells that initially existed in the culture, Luria-Delbrück fluctuation analysis was performed as previously described (Sánchez-Romero and Casadesus, 2014). Briefly, a small number of cells were used to inoculate parallel tubes containing LB broth and were then grown to saturation to obtain equal cell densities. One hundred microliters of overnight samples (~2 × 10^8^ cells) from individual subcultures were separately plated onto LB agar plates containing tigecycline at concentrations of 5 × MIC or 2.5 × MIC. Simultaneously, samples containing the same amount of bacteria from single bacterial cultures were plated onto parallel plates lacking antibiotic. Colonies were counted after 48 h incubation. The concentration of tigecycline was selected to suppress sensitive cells in bacterial cultures and allow the growth of subpopulations containing high-level resistance that was judged by the results of MIC and PAP testing.

Survivors picked up from the drug-containing plates were determined susceptibility to tigecycline by an agar dilution method according to CLSI recommendation M100-S25. After seven daily subcultures in antibiotic-free medium, the tigecycline MICs were re-tested to check the stability of the resistant phenotypes. To determine whether efflux activity was responsible for reduced susceptibility to tigecycline, 2-fold serial broth microdilutions in 96-well plates in the presence or absence of 20 μg/mL of the efflux pump inhibitor phenylalanine-arginine β-naphthylamide (PAβN) dihydrochloride (Sigma, St. Louis, USA). Both unexposed parental strains and survivors showing stable profiles with 4- to 8-fold increases in MICs were chosen for further study. These selections were performed in triplicate and experiments were repeated twice. If the MIC values decreased 4-fold or greater upon exposure to PAβN, efflux activity was judged as high. In order to explore the possibility of cross heteroresistance between unrelated antibiotics, susceptibility to ciprofloxacin was also determined for heteroresistant colonies.

### Accumulation of tigecycline

Efflux activity of selected isolates was further evaluated by determining the accumulation of tigecycline (inversely proportional to efflux activity) according to the method of ciprofloxacin accumulation (Sun et al., 2011). A liquid chromatography assay was used to determine tigecycline concentrations (Li et al., 2004). Differences in drug accumulation at steady-state between randomly selected survivors and the parental strain14028 (see results) were analyzed for statistical significance using a two-tailed Student's *t*-test. *P* < 0.05 was taken as significant. Data shown are means of at least three independent technical replicates (±SEM).

### Quantitative real-time Pcr (qRT-PCR) analysis

The expression levels of *marA, soxS, rob*, and *ramA* regulator genes and efflux components *acrA, acrB, tolC*, and *oqxB* were assessed using qRT-PCR. Bacteria were grown to mid-logarithmic phase and total RNA was extracted by using Trizol reagent (Invitrogen, Carlsbad, CA). RNA yield and quality were measured using a BioPhotometer plus instrument (Eppendorf, Shanghai, China) and cDNA was synthesized from 0.5 mg RNA templates using a PrimeScript RT reagent Kit with gDNA Eraser (Perfect Real Time) according to the manufacturer's instructions (TaKaRa, Dalian, China). qRT-PCR was carried out in an IQ5 thermal cycler (Bio-Rad, Hercules, CA) using SYBR® Premix Ex Taq (TaKaRa, Dalian, China). Primers are listed in the Table S1. PCR conditions were as previously described(Abouzeed et al., 2008; Kehrenberg et al., 2009; Zheng et al., 2009; Wong et al., 2015). qRT–PCR experiments were performed in triplicate using different RNA preparations for confirmation of the results. The 2^−ΔΔCt^ method was used to calculate fold changes of mRNA levels of target genes in selected survivors compared with those observed in their respective parental strains (strains 14028, 14028/pHXY0908, and 14028/Δp52). Expression levels of all pump genes and pump regulators of parental strains were designated to be 1. Only genes whose ratios were ≥2-fold changes (either increased or decreased) were considered statistically significant (Zheng et al., 2011).

### DNA sequence analysis

To detect mutations in regions known to be involved in the regulation of the efflux pump AcrAB-TolC, we amplified *acrR, soxR, marR*, and *ramR* using primers and PCR conditions as described previously (Olliver et al., 2004; Abouzeed et al., 2008). The PCR products were sent for DNA sequencing (company and location). Sequences were compared with those from the GenBank nucleotide database using the BLAST (Basic Local Alignment Search Tool) algorithm (http://www.ncbi.nlm.nih.gov/BLAST).

### Plasmid curing

To confirm the role of genes on the plasmid in tigecycline heteroresistance, the cure of plasmid from a heteroresistant isolate 14028/p#52 was conducted using sodium dodecyl sulfate (SDS) as previously described (Akiyama et al., 2013). Briefly, an overnight culture was diluted 100-fold in LB broth containing 10% SDS and then was incubated overnight at 42°C. Bacterial suspensions were streaked onto LB agar and individual colonies were replica plated onto LB agar plates with or without olaquindox (50 μg/mL). To verify the plasmid loss, PCR amplification of the *oqxB* gene was performed for those colonies that did not grow in olaquindox. The cured strain was named 14028/Δp52. Heteroresistance to tigecycline of 14028/Δp52 were characterized and experiments were conducted as those used for 14028 and 14028/pHXY0908.

## Results

### Acquiring an *oqxAB*-bearing plasmid pHXY0908 makes *Salmonella Enterica* serovar typhimurium heteroresistant to tigecycline

**Figure 1:**
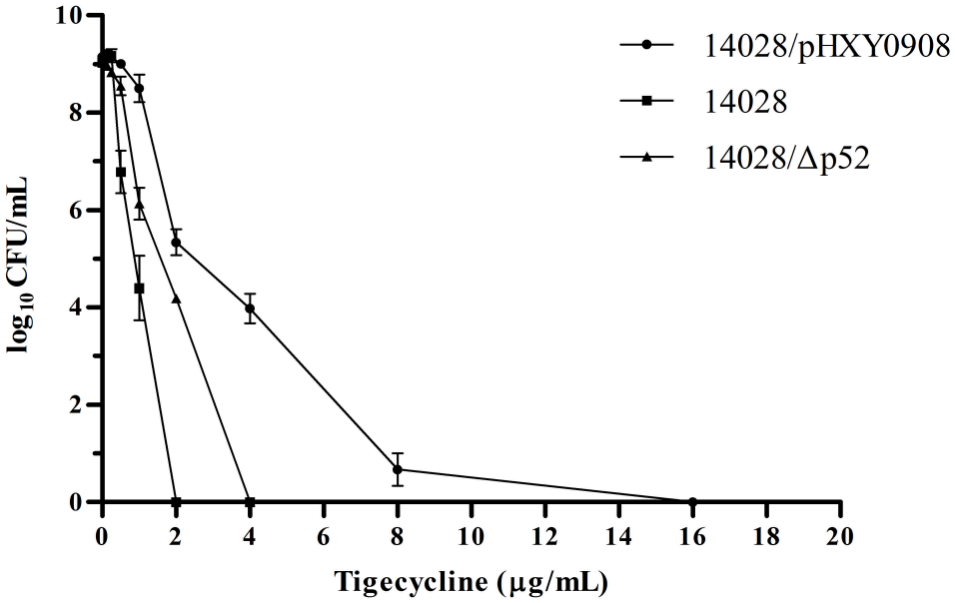
**Susceptibility of *S*. Typhimurium14028, 14028/pHXY0908 and 14028/Δp52 to tigecycline as demonstrated by population analysis**. The *x* axis indicates the tigecycline concentration in micrograms per milliliter used to select supopulations with higher tigecycline-resistance levels, and on the *y* axis, the frequency of bacterial cells is given as the logarithm to the base 10 of cfu per milliliter.

The tigecycline MICs of the 14028, 14028/pHXY0908, and 14028/Δp52 strains did not show significant differences and were 0.5, 1, and 1 μg/mL, respectively. However, in strain 14028/pHXY0908, the PAP curve indicated the presence of subpopulations with high-level resistance to tigecycline at frequencies ranging from 10^−5^ to 10^−8^. Conversely, subpopulations of 14028 and 14028/Δp52 did not show this extent of resistance and grew to less than four times their MICs (Figure 1).

### pHXY0908 increases the frequency of emergence of strains with reduced susceptibility

Luria-Delbrück fluctuation analysis indicated that bacteria survived the selection process at relatively low frequencies (≤10^−7^; Tables S2–S4). The strain 14028/pHXY0908 survivors were cells that were present in the starting bacterial culture indicating no selection occurred during this experiment. MIC of isolates directly picked from drug-containing plates showed widely distributed values (Figure 1S).

We re-tested susceptibility of these colonies by subculturing in antibiotic-free medium. A substantial number of these strains returned to tigecycline susceptibility (Figure 1S). Only 4 (4/43), 21 (21/55), and 9 (9/36) isolates of 14028, 14028/pHXY0908, and 14028/Δp52 respectively, showed stable profiles with 4 to 8-fold MIC increases.

The frequency of emergence of these isolates was significantly higher in strain 14028/pHXY0908 than in either 14028 or 14028/Δp52 (*P* < 0.05). Ciprofloxacin susceptibility of 21 heteroresistant isolates of 14028/pHXY0908 were tested and exhibited 4- to 8-fold increases in MICs (1–2 μg/mL for heteroresistant isolates; 0.25 μg/mL for 14028/pHXY0908) (detailed data not shown).

### Tigecycline susceptibility was reversed by adding efflux pump inhibitor (PAβN)

As efflux pumps are implicated in tigecycline resistance in *Salmonella*, we examined whether efflux pumps were also associated with tigecycline heteroresistance. The addition of the efflux pump inhibitor PAβN reduced MIC values 2- to 64-fold (Table S5). This data indicated that efflux pumps are involved in heteroresistance.

### Less accumulation of tigecycline in strains with reduced susceptibility

**Figure 2:**
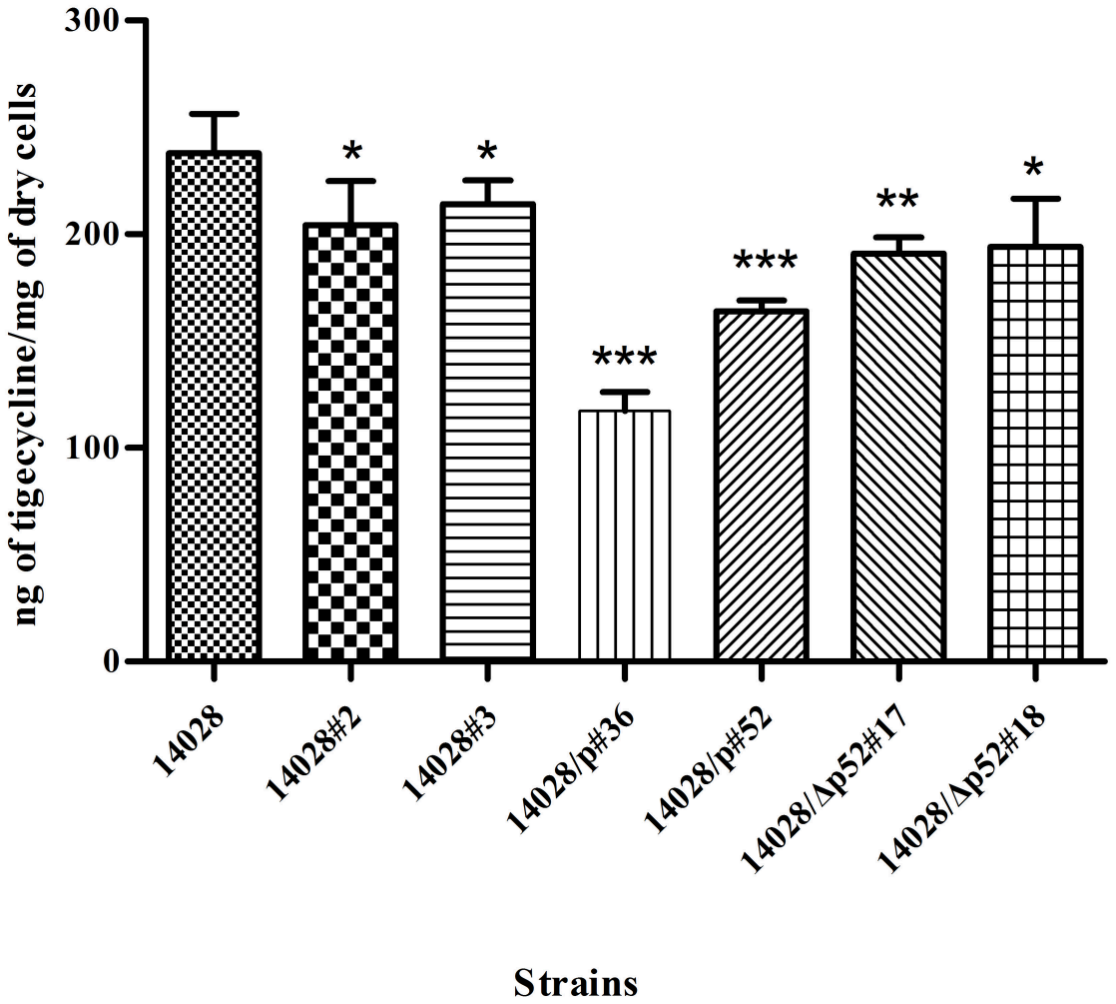
**Accumulation of tigecycline in isolates relative to strain 14028**. The data shown are the means of two biological replicates. ^*^P < 0.05; ^**^P < 0.01; ^***^P < 0.001.

Since the intracellular accumulation of drugs were inversely proportional to efflux activity, we measured concentrations of tigecycline in selected survivors and parental strain. Results showed that the steady-state tigecycline concentrations in two plasmid containing strains (14028/p#36 and 14028/p#52) were 2- and 3-fold less than the parental strain 14028 (*P* < 0.001). Reduced tigecycline accumulation was also observed in 14028#2 (*P* < 0.05), 14028#3(*P* < 0.05), 14028/Δp52#17 (*P* < 0.01), and 14028/Δp52#18 (*P* < 0.05) (Figure 2).

### Upregulation of pump genes and regulators in strains with reduced susceptibility

**Figure 3:**
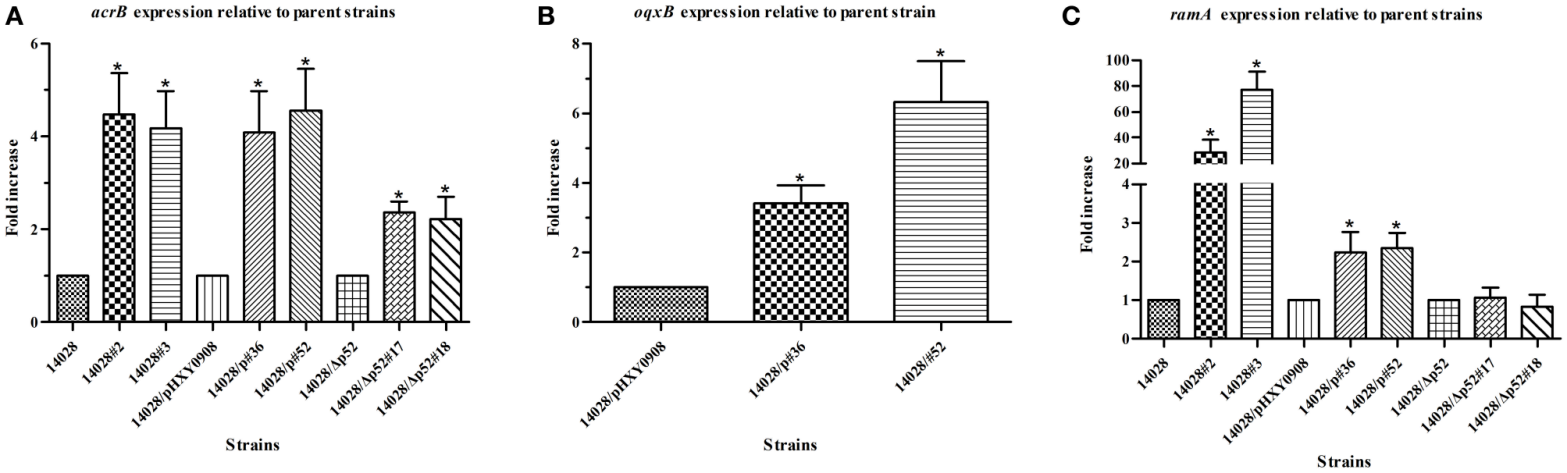
**Comparison of relative expression levels of *acrB* (A), *oqxB* (B) and *ramA* (C) in isolates 14028#2, 14028#3, 14028/p#36, 14028/p#52, 14028/Δp52#17 and 14028/Δp52#18 compared with their respective parental strains 14028, 14028/pHXY0908 and 14028/Δp52, and *oqxB* (B) in isolates 14028/p#36 and 14028/p#52 compared with 14028/pHXY0908**. Expression level of *acrB, oqxB*, and *ramA* of parental strains were designated to be 1. ^*^P < 0.01.

To further confirm that efflux pumps were responsible for the reduced tigecycline susceptibility and accumulation, we selected survivors and determined the relative mRNA levels of the efflux pump genes *acrB* and *oqxB*. The levels of both genes in all strains were 2- to 5-fold greater than controls. This trend was also seen with the global regulator *ramA*. Interestingly, in two isolates the increases were 28.5- and 77-fold (Figure 3 and Figure 2S). Up-regulation of pump genes *acrA, tolC*, and pump regulator *marA* were observed in all of tested isolates. Strain 14028/p#36 also showed increased expression of *soxS* while *rob* expression was similar to those of the parental strains.

Interestingly, curing the plasmid in 14028/p#52 decreased the expression of *acrA* by −5.45- ± 0.70-fold and *acrB* by −3.47- ± 0.57-fold (data not shown) and decreased the tigecycline MIC to 1 μg/mL.

### Mutations in *ramR* gene leads to overexpression of *ramA* and *acrAB*

To investigate causes of the increased expression of *ramA* and *acrAB*, DNA sequencing of the regulatory regions was performed. Analyzing of *ramR* gene detected a point mutation, ACC → CCC, at position 52 leading to the amino acid substitution of threonine for proline (T18P) in the resistant isolates 14028/p#36, 14028/p#52, 14028/Δp52#17, and 14028/Δp52#18 (Table 2). In 14028#2, a mutation at amino acid 39 of RamR (V39A) was identified. An insertion of 1bp (A) located at nucleotide position 63 within the *ramR* gene was identified in 14028#3. This insertion led to occurrence of a premature stop codon (R21Stop). All of tested strains were confirmed to have wild type *acrR, soxR*, and *marR* sequences.

**Table 2:** **Characteristics of *Salmonella* strains identified in this study**.

**Strains**	**Heteroresistance^a^**	**MIC range (μg/mL)^b^**	**Stability rate^c^ (%)**	**Inhibited by PAβN^d^**	**Intracellular accumulation^e^**	**Expression level of *acrB*, *oqxB*, *ramA*^f^**	**Nucleotide Mutation (amino acid change)^g^**
14028	−	0.5–2	4/43 (9.30%)	Yes	^***^	↑	T116C (V39A)^g1^
							63insA1(R21Stop)^g2^
14028/pHXY0908	+	1–8	21/55 (38.18%)	Yes	^*^	↑	A52C (T18P)^g3^
14028/Δp52	−	1–8	9/36 (25%)	Yes	^**^	↑	A52C (T18P)^g3^

a , +, heteroresistance to tigecycline, -, homogeneous response to tigecycline;

b , MICs of isolates directly picked up from drug-containing plates in the performance of Luria-Delbrück fluctuation analysis;

c , frequencies of emergence of isolates showing stable profiles with 4 to 8-fold increases in MICs;

d , restoration of tigecycline susceptibility by inhibition of the pump with PAβN;

e *, intracellular accumulation of tigecycline of selected survivors compared with 14028*,

*** *, maximum*,

** *, medium*,

* , minimum;

f , ↑, upregulation of acrB and ramA in survivors compared with those observed in their respective parental strains 14028, 14028/pHXY0908 and 14028/Δp52, and of oqxB in 14028/p#36 and 14028/p#52;

g *, nucleotide mutation and amino acid substitution in RamR, g1, mutation found in 14028#2, g2, mutation found in 14028#3, g3, mutation found in 14028/p#36, 14028/p#52, 14028/Δp52#17, and 14028/Δp52#18, ins, insertion*.

## Discussion

Heteroresistance describes the coexistence of susceptible and resistant isolates in an otherwise genetically identical population (Hung et al., 2012). This study illustrates that heteroresistance to tigecycline in *S*. Typhimurium harboring an *oqxAB*-bearing multidrug resistance plasmid (Table 2). The increase in resistant subpopulations was greatest in strain 14028/pHXY0908 compared to strains 14028 and 14028/Δp52. It also exhibited a heteroresistance profile while a homogeneous response to tigecycline was observed in parental strain 14028. Interestingly, although 14028/Δp52 and 14028/pHXY0908 exhibited the same susceptibility to tigecycline by the broth microdilution method, subpopulations of 14028/Δp52 were unable to survive at 4 × MIC, similar to 14028. This indicated that the *oqxAB*-bearing plasmid is the reason for the heteroresistant phenotype of 14028/pHXY0908.

Subpopulations with high-level resistance to tigecycline occurred at relatively low frequencies (10^−5^–10^−8^) and MIC-values (1 μg/mL) were below the susceptibility breakpoint (2 μg/mL). This indicates that the CLSI reference methods for antimicrobial susceptibility testing using an inoculum of 5 × 10^5^ CFU/mL would classify such heteroresistant isolates as susceptible (Gomes et al., 2015). A similar assessment of susceptibility levels were found in a previous study that identified heterogeneous growth to carbapenems in *K. pneumoniae* carbapenemase (KPC)-producing *Enterobacteriaceae* (Pournaras et al., 2010). Accordingly, evaluating results of susceptibility testing should include determination of heteroresistant isolates.

In the present study, a resistance profile was maintained in the absence of selection stress in *S*. Typhimurium. The frequencies of emergent isolates showing stable profiles (4- to 8-fold increase in MIC) were significantly higher in 14028/pHXY0908 than in 14028 and 14028/Δp52. Again, this suggests that plasmid pHXY0908 facilitates the appearance of tigecycline-non-susceptible isolates. Heteroresistant isolates of 14028/pHXY0908 also had a decreased susceptibility to ciprofloxacin. This indicated the presence of a mechanism leading to cross-heteroresistance to different antibiotic classes (Hornsey et al., 2010).

Previous study has indicated that upregulation of the AcrAB efflux pump upon exposure to ciprofloxacin caused cross-resistance to tigecycline (Hornsey et al., 2010). This occurrence is of great concern because of the greater probability to induce cross-resistance to new antibiotics with the application of empirical therapies. Overexpression of efflux pump can also lead to tigecycline resistance in *S. aureus* and, furthermore, that the acquisition of this resistance may be associated with reduced susceptibility to vancomycin (Herrera et al., 2016). After addition of PAβN, reduction of MIC values was observed for all resistant isolates that indirectly proves that efflux pump over-expression contributed to reduced tigecycline susceptibility. Furthermore, we directly confirmed efflux activity by determining the accumulation of intracellular of tigecycline. Therefore, this was an effective mechanism for heteroresistance or non-susceptibility in *S*. Typhimurium pertaining to activity of tigecycline.

A recent study has been sought to elucidate the roles of *Salmonella* multidrug efflux pumps and AcrAB regulators in tigecycline resistance, and results obtained showed that deletion of *acrAB* led to strains with significantly increased susceptibility to tigecycline and plasmids carrying the *acrAB* restored increased susceptibility of the *acrAB* -deleted mutant (Horiyama et al., 2011). That study demonstrated that reduced susceptibility to tigecycline might develop as a result of increased expression of efflux pump AcrAB in *Salmonella enterica* (Horiyama et al., 2011). Up-regulation of AdeABC, another resistance-nodulation-division efflux pump, has been proved resulting in decreased susceptibility to tigecycline in *A. calcoaceticus–A. baumannii* (Ruzin et al., 2007). In the present study, overexpression of *acrAB* in 14028/p#36 and 14028/p#52 were observed indicating that an active efflux system was closely associated with heteroresistance to tigecycline. Consistent with the published data that AcrAB confers resistance to tigecycline (Horiyama et al., 2011), upregulation of AcrAB was also observed in 14028/Δp52#17 and 14028/Δp52#18. In addition, overexpression of the *oqxB* gene in both heteroresistant isolates indicated that efflux pump OqxAB might play a role in mediating tigecycline heteroresistance in *S*. Typhimurium strains.

Up-regulation of global regulators *ramA, marA*, and *soxS*, which activate the AcrAB efflux pump, were also observed in isolates tested individually or as a group. Additionally, in strains14028/p#36, 14028/p#52, 14028/Δp52#17, and 14028/Δp52#18 we found an amino acid change (T18P) in *ramR*, which has been reported to play a role in the upregulation of RamA and AcrAB (Abouzeed et al., 2008). This resulted in an efflux-mediated MDR phenotype in *S*. Typhimurium.

Overexpression of *ramA* and inactivation of *ramR* involved in AcrAB-dependent tigecycline resistance in *Salmonella* have been suggested previously (Horiyama et al., 2011). In the current study, these mutations in the *ramR* gene led to upregulation of *ramA* resulting in tigecycline heteroresistance in our tested isolates. In *K. pneumoniae, oqxAB* is downregulated by a repressor (*oqxR*) in the presence of a positive level of control *via* RamA, SoxS, and RarA (Veleba et al., 2012; Jiménez-Castellanos et al., 2016). Loss of OqxR repressor function was observed from the plasmid-borne *oqxAB* gene (Wong et al., 2015). Thus, *ramA* overexpression seemed to upregulate the oqxAB efflux pump in strains 14028/p#36 and 14028/p#52. However, further work is necessary to explore whether RamA can control the expression of the plasmid-encoded *oqxAB* gene or whether RamA-mediated efflux pump overexpression can increase plasmid-mediated resistance (Jiménez-Castellanos et al., 2016).

Interestingly, when we cured the *oqxAB*-bearing plasmid from a heteroresistant isolate (14028/p#52), restoration of tigecycline susceptibility and downregulation of pump genes *acrA* and *acrB* were observed (data not shown). After we have test the susceptibility of a cured strain in which the plasmid has been re-introduced and the MIC was 8 μg/mL, indicating that approach used for curing the plasmid would not affect the susceptibility to tigecycline (data not shown). Thus, we hypothesized that in addition to the global transcriptional regulator RamA, genes on the plasmid might be also involved in the regulation of AcrAB-TolC, contributing to tigecycline heteroresistance. Deciphering potential mechanisms by which the *oqxAB*-bearing plasmid participates in the regulation of AcrAB-TolC pump are under the investigation in our laboratory.

It remains controversial whether heteroresistance is directly linked to treatment failure (Moore et al., 2003; Falagas et al., 2008; Deresinski, 2009; Lee et al., 2011) even though there is clinical evidence that tigecycline monotherapy is associated with a high mortality rate. Results obtained in the present study suggested that the rapid induction of efflux pumps AcrAB-TolC and OqxAB upon a single exposure to tigecycline in a heteroresistant *Salmonella* strain *in vitro* might leads to tigecycline resistance. Thus, heteroresistance should be taken into account when tigecycline treatment is used to avoid inadvertently inducing bacterial resistance. Appropriate detection methods are required to guide therapeutic decision-making.

Heteroresistance to tigecycline has not been previously reported so it is unclear whether there are other heteroresistant species and what clinical significance this may have. Systematic investigations on tigecycline heteroresistance in other pathogens and the relationship between heteroresistance and treatment failure are of practical importance.

In summary, the observations of present study suggested that an epidemic *oqxAB*-bearing IncHI2-type plasmid might engender a heteroresistant phenotype to *S*. Typhimurium against tigecycline. This phenomenon was probably related to overexpression of multidrug resistant efflux pumps AcrAB-TolC and OqxAB.

## Author contributions

YC and JS designed the experiments, analyzed data, and wrote the manuscript. YC and DH performed the experiments and collected the data. YC, QZ, XL, and JS prepared the manuscript. All authors have contributed to, seen and approved the manuscript.

### Conflict of interest statement

The authors declare that the research was conducted in the absence of any commercial or financial relationships that could be construed as a potential conflict of interest.
