# Supplementary material for: Efflux Pump Overexpression Contributes to Tigecycline Heteroresistance in Salmonella enterica serovar Typhimurium
Source: Front Cell Infect Microbiol. 2017 Feb 17;7:37. doi: 10.3389/fcimb.2017.00037 (PMC5313504; doi:10.3389/fcimb.2017.00037)
Supplement: Supplementary file 1 [file Table1.docx]

**Table S1.** Primers used in this study.

| Gene | Oligonucleotides (5΄→3΄) | Reference |
| --- | --- | --- |
| RT-PCR expression analysis |  |  |
| *acrA* | F: TGTGGAACCAGTAATGCCGTC | Kehrenberg et al., 2009 |
|  | R: GAAGCAGGAGCTGGCAAATG |  |
| *acrB* | F: GGCATTGGGTATGACTGGAC | Zheng et al., 2009 |
|  | R: GCATTACGGAGAACGGGATAG |  |
| *tolC* | F: TGCCGCAACTGGGTTTAGG | Kehrenberg et al., 2009 |
|  | R: CAGAGAAGCGCTGGTTTCATTG |  |
| *oqxB* | F: TATCTCATTGGCGGCGTGAA | Wong et al., 2015 |
|  | R: CGCGATTTTGGCGTTGATCT |  |
| *ramA* | F: TTTCCGCTCAGGTTATCGAC | Zheng et al., 2009 |
|  | R: CGGGCAATATCATCAATACG |  |
| *marA* | F: ATTCTCTATCTGGCGGAAC | Zheng et al., 2009 |
|  | R: CGGGTCAATGTTTGCTGTG |  |
| *soxS* | F: AAATCGGGCTACTCCAAG | Zheng et al., 2009 |
|  | R: TACTCGCCTAATGTTTGATG |  |
| *robA* | F: TATTCCGCCAGTGCTTTATG | Zheng et al., 2009 |
|  | R: CCTGCTCATCGTCTTTCTCC |  |
| *rrsG* | F: GTTACCCGCAGAAGAAGCAC | Zheng et al., 2009 |
|  | R: CACATCCGACTTGACAGACC |  |
| Detection of mutations |  |  |
| *ramR* | F: CGTGTCGATAACCTGAGCGG | Abouzeed et al., 2008 |
|  | R: AAGGCAGTTCCAGCGCAAAG |  |
| *marR* | F: CTGTCCTGATCTGGCGAAAA | Zheng et al., 2009 |
|  | R: GAACCCGGCGTAGAGTGAT |  |
| *soxR* | F: TCATCGCCTGGCTACAACAA | Zheng et al., 2009 |
|  | R: GCGGCGCTTTAGTTTTAGGTG |  |
| *acrR* | F: TGTGACAACCCCAACTTC | Zheng et al., 2009 |
|  | R: AATATCACGCCTGTAACAAAC |  |
