# Supplementary material for: Efflux Pump Overexpression Contributes to Tigecycline Heteroresistance in Salmonella enterica serovar Typhimurium
Source: Front Cell Infect Microbiol. 2017 Feb 17;7:37. doi: 10.3389/fcimb.2017.00037 (PMC5313504; doi:10.3389/fcimb.2017.00037)
Supplement: Supplementary file 2 [file Table2.docx]

**Table S2.** Fluctuation in the frequencies of tigecycline-resistant isolates obtained upon plating 10^8^ colony-forming-units of 14028/pHXY0908 on LB + tigecycline (5 μg/mL).

| Number of colonies | Individual cultures | Single culture |
| --- | --- | --- |
|  | 12 | 7 |
|  | 8 | 1 |
|  | 1 | 2 |
|  | 6 | 1 |
|  | 6 | 3 |
|  | 8 | 1 |
|  | 10 | 2 |
|  | 6 | 1 |
|  | 8 | 3 |
|  | 3 | 3 |
|  | 5 | 1 |
|  | 1 | 1 |
|  | 22 | 2 |
|  | 7 | 3 |
|  | 8 | 2 |
|  | 7 | 1 |
|  | 2 | 1 |
|  | 3 | 6 |
|  | 2 | 5 |
|  | 2 | 2 |
| Average | 6.35 | 2.4 |
| SD | 4.82 | 1.76 |
| Coefficient of variation | 0.76 | 0.73 |
