# Supplementary material for: Efflux Pump Overexpression Contributes to Tigecycline Heteroresistance in Salmonella enterica serovar Typhimurium
Source: Front Cell Infect Microbiol. 2017 Feb 17;7:37. doi: 10.3389/fcimb.2017.00037 (PMC5313504; doi:10.3389/fcimb.2017.00037)
Supplement: Supplementary file 3 [file Table3.docx]

**Table S3.** Fluctuation in the frequencies of tigecycline-resistant isolates obtained upon plating 10^8^ colony-forming-units of 14028 on LB + tigecycline (1.25 μg/mL).

| Number of colonies | Individual cultures | Single culture |
| --- | --- | --- |
|  | 12 | 5 |
|  | 9 | 11 |
|  | 17 | 7 |
|  | 6 | 5 |
|  | 30 | 5 |
|  | 9 | 18 |
|  | 0 | 10 |
|  | 4 | 10 |
|  | 0 | 19 |
|  | 4 | 6 |
|  | 2 | 14 |
|  | 7 | 10 |
|  | 19 | 25 |
|  | 2 | 18 |
|  | 2 | 9 |
|  | 24 | 26 |
|  | 29 | 4 |
|  | 1 | 11 |
|  | 21 | 10 |
|  | 1 | 18 |
| Average | 9.95 | 12.05 |
| SD | 9.91 | 6.59 |
| Coefficient of variation | 1.00 | 0.55 |
