# Supplementary material for: Efflux Pump Overexpression Contributes to Tigecycline Heteroresistance in Salmonella enterica serovar Typhimurium
Source: Front Cell Infect Microbiol. 2017 Feb 17;7:37. doi: 10.3389/fcimb.2017.00037 (PMC5313504; doi:10.3389/fcimb.2017.00037)
Supplement: Supplementary file 4 [file Table4.docx]

**Table S4.** Fluctuation in the frequencies of tigecycline-resistant isolates obtained upon plating 10^8^ colony-forming-units of 14028/Δp52 on LB + tigecycline (2.5 μg/mL).

| Number of colonies | Individual cultures | Single culture |
| --- | --- | --- |
|  | 12 | 3 |
|  | 4 | 0 |
|  | 9 | 5 |
|  | 3 | 11 |
|  | 1 | 13 |
|  | 1 | 5 |
|  | 1 | 4 |
|  | 20 | 12 |
|  | 1 | 9 |
|  | 3 | 5 |
|  | 5 | 7 |
|  | 12 | 1 |
|  | 4 | 1 |
|  | 3 | 2 |
|  | 2 | 2 |
|  | 3 | 1 |
|  | 2 | 32 |
|  | 4 | 8 |
|  | 4 | 3 |
|  | 11 | 1 |
| Average | 5.25 | 6.25 |
| SD | 5.00 | 7.23 |
| Coefficient of variation | 0.95 | 1.16 |
