# Supplementary material for: Efflux Pump Overexpression Contributes to Tigecycline Heteroresistance in Salmonella enterica serovar Typhimurium
Source: Front Cell Infect Microbiol. 2017 Feb 17;7:37. doi: 10.3389/fcimb.2017.00037 (PMC5313504; doi:10.3389/fcimb.2017.00037)
Supplement: Supplementary file 5 [file Table5.docx]

**Table S5.** The MIC of tigecycline (TIG) of strains isolated in the Luria-Delbrück fluctuation analysis and parental strains 14028, 14028/p and 14028/Δp52 with or without an efflux pump inhibitor, Phe-Arg-β-naphtylamide (PAβN).

| Isolates | MIC (μg/mL) | |
| --- | --- | --- |
|  | TIG | TIG+PAβN |
| **14028** | 0.5 | 0.25 |
| 14028#2 | 2 | 0.5 |
| 14028#3 | 2 | 0.5 |
| 14028#5 | 2 | 0.5 |
| 14028#6 | 2 | 0.5 |
| **14028/pHXY0908** | 1 | 0.125 |
| 14028/p#1 | 8 | 0.25 |
| 14028/p#2 | 8 | 0.25 |
| 14028/p#3 | 8 | 0.25 |
| 14028/p#4 | 8 | 0.25 |
| 14028/p#12 | 8 | 0.25 |
| 14028/p#25 | 8 | 0.5 |
| 14028/p#26 | 8 | 0.25 |
| 14028/p#27 | 8 | 0.125 |
| 14028/p#28 | 8 | 0.25 |
| 14028/p#29 | 8 | 0.25 |
| 14028/p#30 | 8 | 0.125 |
| 14028/p#31 | 8 | 0.25 |
| 14028/p#32 | 8 | 0.125 |
| 14028/p#33 | 8 | 0.125 |
| 14028/p#34 | 8 | 0.125 |
| 14028/p#36 | 8 | 0.25 |
| 14028/p#42 | 4 | 0.125 |
| 14028/p#47 | 8 | 0.25 |
| 14028/p#52 | 8 | 0.25 |
| 14028/p#53 | 8 | 0.25 |
| 14028/p#55 | 4 | 0.5 |
| **14028/Δp52** | 1 | 0.25 |
| 14028/Δp52#12 | 4 | 0.5 |
| 14028/Δp52#17 | 8 | 1 |
| 14028/Δp52#18 | 8 | 1 |
| 14028/Δp52#20 | 8 | 1 |
| 14028/Δp52#23 | 4 | 0.5 |
| 14028/Δp52#30 | 8 | 0.5 |
| 14028/Δp52#33 | 4 | 1 |
| 14028/Δp52#34 | 4 | 0.5 |
| 14028/Δp52#35 | 4 | 0.5 |
