# Supplementary figures and images for: Efflux Pump Overexpression Contributes to Tigecycline Heteroresistance in Salmonella enterica serovar Typhimurium
Source: Front Cell Infect Microbiol. 2017 Feb 17;7:37. doi: 10.3389/fcimb.2017.00037 (PMC5313504; doi:10.3389/fcimb.2017.00037)

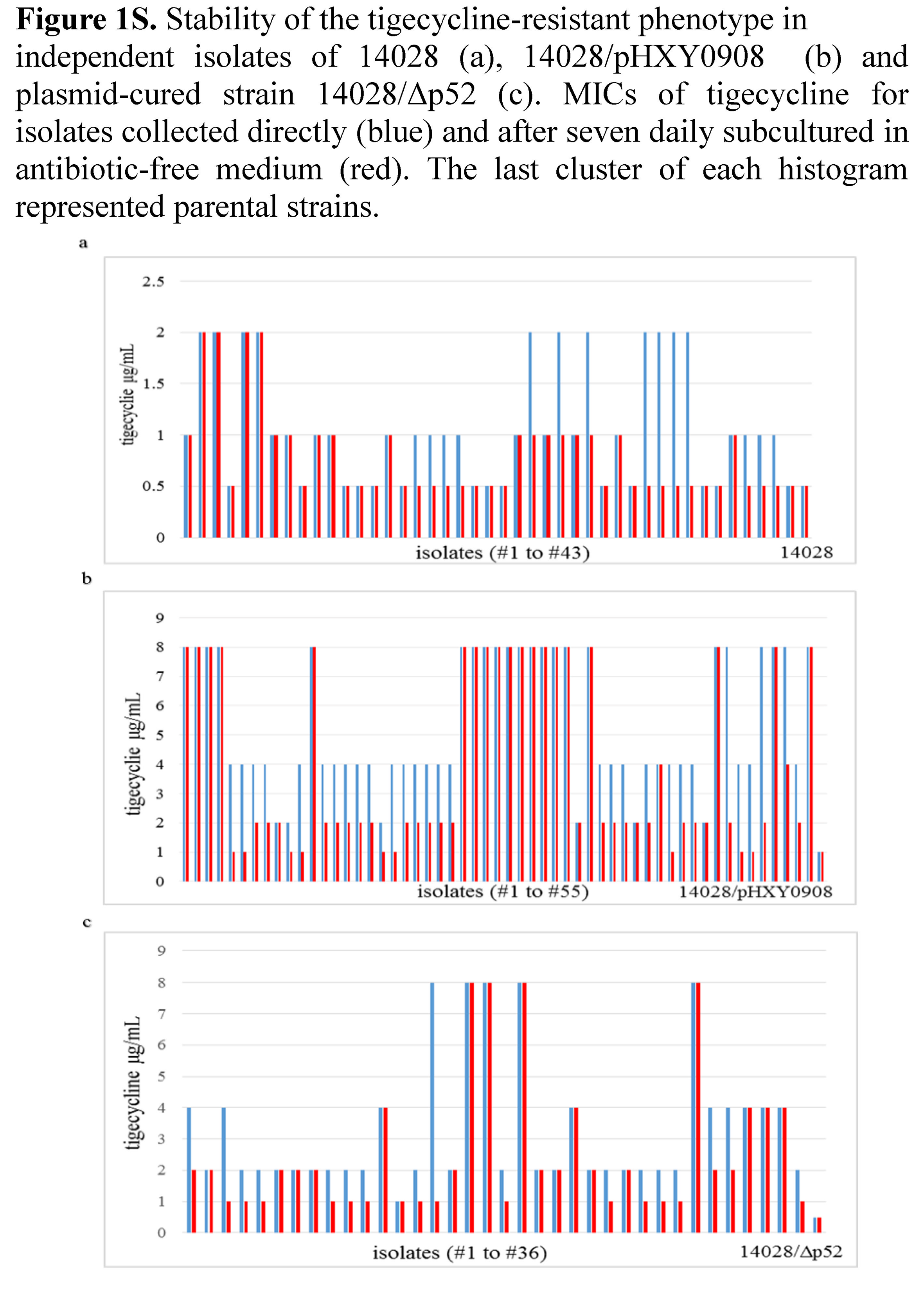

Supplement: Supplementary file 6 [file Image1.tif]

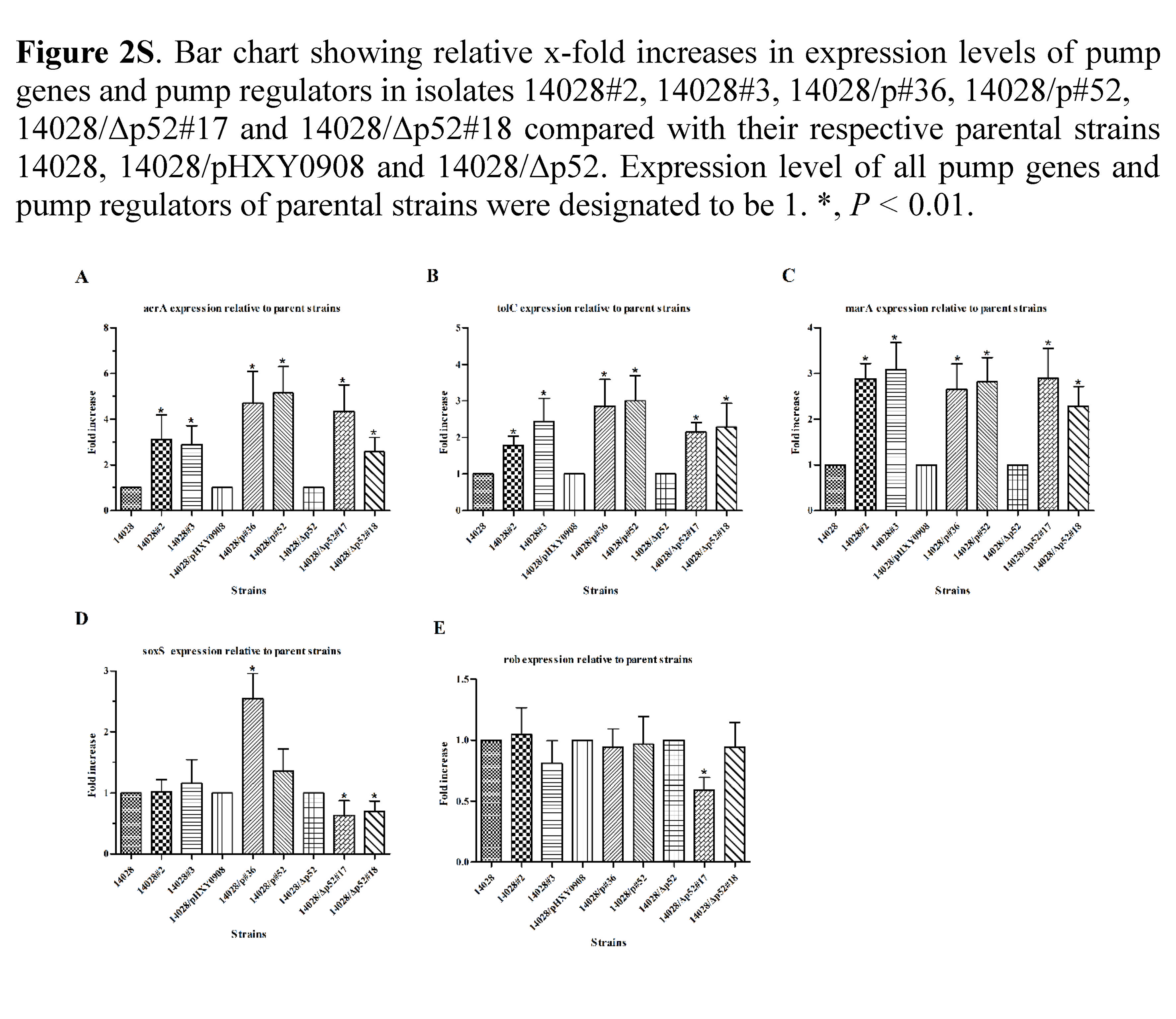

Supplement: Supplementary file 7 [file Image2.tiff]
